# Incidence and correlates of receiving cigarettes as gifts and selecting preferred brand because it was gifted: Findings from the ITC China Survey

**DOI:** 10.1186/1471-2458-12-996

**Published:** 2012-11-17

**Authors:** Li-Ling Huang, James F Thrasher, Yuan Jiang, Qiang Li, Geoffrey T Fong, Anne CK Quah

**Affiliations:** 1 Department of Health Promotion, Education & Behavior, Arnold School of Public Health, University of South Carolina, Columbia, SC, 29208, USA; 2 Center for Population Health Research, National Institute of Public Health, Cuernavaca, Morelos, Mexico; 3 National Tobacco Control Office, Chinese Center for Disease Control and Prevention, Beijing, China; 4 Department of Psychology, University of Waterloo, Waterloo, ON, Canada; 5 Ontario Institute for Cancer Research, Toronto, ON, Canada

**Keywords:** Tobacco, Cigarette gifting, Preferred cigarette brand

## Abstract

**Background:**

Giving cigarettes as gifts is a common practice in China, but there have been few systematic studies of this practice. The present study was designed to estimate the incidence of receiving cigarettes as gifts, correlates of this practice, and its impact on brand selection in a representative sample of urban adult smokers in China.

**Methods:**

Data were analyzed from Wave 2 of the International Tobacco Control (ITC) China Survey, where 4843 adult urban smokers were interviewed in six major Chinese cities between October 2007 and January 2008. The incidence of most recent cigarette acquisition due to gifting and the prevalence of preferred brand selection due to having received it as a gift were estimated. Bivariate and adjusted logistic regression models were estimated to identify factors associated with these two outcomes.

**Results:**

The incidence of receiving cigarettes as a gift at most recent cigarette acquisition was 3.5%. Smokers who received these gifted cigarettes were more likely to be female, older, have higher educational attainment, live in Beijing, and smoke fewer cigarettes per day. The prevalence of choosing one’s preferred brand due to having received it as a gift was 7.0%, and this was more likely among smokers who lived in Beijing and Guangzhou, had lower educational attainment, smoked less frequently, and had smoked their preferred brand for less than one year.

**Conclusions:**

The 3.5% incidence of one’s most recent cigarette acquisition due to gifting is consistent with prevalence estimates based on longer reference periods and translates into the average smoker receiving a gift of cigarettes approximately five times a year. Gifting also appears to have a significant influence on brand preference. Tobacco control interventions in China may need to denormalize the practice of giving cigarettes as gifts in order to decrease the social acceptability of smoking.

## Background

China is the world’s largest producer and consumer of tobacco, with about 301 million smokers and about one million smoking-related deaths every year [1,2]. Smoking is socially acceptable, particularly among males, over half of whom are smokers (52.9% compared to 2.4% of women [2]). The gifting and exchange of cigarettes among Chinese men appear common, and the tobacco industry has actively promoted it [3,4]. Nevertheless, previous research on this topic has mostly been conducted with weak designs and measurement approaches. The current research draws from representative samples of urban Chinese smokers to better understand the frequency and impact of this practice.

In Chinese society, gifting cigarettes facilitates relationship building across a variety of daily interpersonal interactions and special social occasions [3,4]. Transnational and domestic tobacco companies have actively exploited this custom to build brand identity while reinforcing this practice [3]. Although a few studies have called attention to cigarette gifting in China, methodological issues, such as non-representative samples, have impeded understanding of how widespread this custom is and who practices it.

Gifts are a medium for establishing and maintaining interpersonal relationships in Chinese culture [5]. *Guanxi* (i.e., relationships and connections), *renqing* (i.e., feelings and social favors), *mianzi* (i.e., face and respect), and *bao* (i.e., reciprocity) are all dominant Chinese values that give meaning to gift-giving behavior and its role in maintaining harmonious social bonds and interaction [4-11]. The nature and value of gifts range widely from an inexpensive bag of food to premium cigarettes based on the relative social status of the giver and recipient, previous gifting interactions between parties, and expectations of reciprocity from the recipient [3,11].

Cigarettes are popular gifts [3,4,6,9,12]. The value and class of the cigarette gift are influenced by the social status and intentions of the two parties involved [4,13]. Because the price of cigarette brands ranges widely, from $0.14 (1 RMB) up to $107 (765 RMB) per pack [14,15]. Recipients can easily determine the monetary value of cigarette gifts and provide suitable reciprocation in the future [4,16]. Transnational tobacco companies have strategically priced their products as premium gifts in order to fit into Chinese cigarette gifting customs, and they have promoted their products with culturally attractive packaging to compete with local premium brands [3].

The gifting of expensive premium cigarettes displays affluence and status, facilitates business deals, and helps move through government bureaucracies [3,4,14,17]. Among friends and business partners, gifts of expensive cigarettes are used to gain face, to show respect and hospitality, and to build friendship and guanxi [3,17,18]. Businessmen, government officials, and doctors appear particularly subject to cigarette gifting norm [3,13,17]. A convenience sample of 103 male physicians indicated that 84% of male doctor smokers and 29% of male doctor nonsmokers always or sometimes accepted cigarettes as gifts [19]. Doctors feel obliged to accept cigarette gifts from patients’ family members because they do not want to be seen as indifferent to the patient [13].

Gifting cigarettes appears most prevalent during Lunar New Year celebrations [3,18]. A 2010 survey in Jiangsu province indicated that over 50% of 1200 respondents reported that they planned to give cigarettes as gifts during the Lunar New Year [18]. A 2010 street intercept survey of 528 Shenyang residents found similar results, with about 47% reporting that they had ever given cigarettes as gifts and about 56% reporting that they planned to give cigarette gifts during the Lunar New Year, despite the fact that 79% acknowledged that cigarette gifts represented a health hazard [20]. These data are consistent with a 1989 Philip Morris study showing that most 15-to-60-year-old Shanghai (53%) and Beijing (60%) residents had received foreign cigarettes as gifts in the previous three months, which included the Lunar New Year [21,22]. The results illustrate the popularity of foreign cigarettes for gifts when considering the small segment of the Chinese market that foreign brands comprised at that time (less than 5%).

As in other countries [23,24], receipt of cigarettes and cigarette accessories as gifts in China appears to promote youth smoking uptake [25-28]. Furthermore, Chinese smokers reported that cigarette gifting and sharing hindered their cessation efforts [16,29]. Aside from these studies of the relationship between gifting and both quitting and uptake, the only other study of the correlates of cigarette gifting comes from a 2002 cross-sectional market survey of 2042 residents in four cities (Beijing, Shanghai, Guangzhou, and Chengdu), in which 32% of respondents reported that they had given cigarettes as gifts to others in the previous six months (which included the Lunar New Year) [30]. Cigarette gift givers were younger, had higher educational attainment and personal income, worked in private companies, and worked in sales or management positions [30]. Cigarette gifts were given mostly to friends and colleagues (61%), bosses and supervisors (14%), business partners (13%), and family (10%) [30].

Existing studies of cigarette gifting in China primarily involve convenience samples and have measurement issues that impede a clear understanding of the frequency of this custom in the general population and of the population segments that most often practice it. This study reports findings from a population-based sample of Chinese smokers in major metropolitan areas in order to better estimate the incidence, correlates, and impact of this practice.

## Methods

### Study sample

Data were analyzed from Wave 2 of the International Tobacco Control (ITC) China Survey, a population-based representative sample of adult smokers surveyed between October 2007 and January 2008 in six Chinese cities (i.e., Beijing, Shanghai, Guangzhou, Shenyang, Changsha and Yinchuan). A stratified multi-stage cluster sampling design was used to produce representative samples of 800 smokers and 200 nonsmokers within each city (see Wu et al. [31] for greater detail). The analytic sample for the current study included only adult smokers, who had smoked at least 100 cigarettes in their lifetime and were smoking at least once a week at the time of the Wave 2 survey. The Wave 2 participants included both Wave 1 participants who were successfully re-contacted (n=3863; 81.6% retention) and new participants to replenish those lost to follow-up (n=980). This replenishment sample drew from households that were enumerated in Wave 1 but were not surveyed at that time.

All materials and procedures used in the ITC China Survey were reviewed and cleared for ethics by the Office of Research at the University of Waterloo (Waterloo, Canada), and the Institutional Review Boards at Roswell Park Cancer Institute (Buffalo, USA), the Cancer Council Victoria (Victoria, Australia), and the China National Centers for Disease Control and Prevention (Beijing, China).

### Measurements

#### Cigarettes received as a gift

Two questions were used to assess cigarette gifting. Smokers were asked to report the source of the cigarettes that they had most recently obtained, using a list of 16 sources, including as a gift, from supermarkets, convenience stores, gas stations, restaurants, entertainment venues (bars or coffee/tea shops), hotels, duty-free shops, tobacco shops, military stores, vending machines, street venders, venders on trains or ships, overseas, internet, and other sources. After indicating the brand that they smoked most often in the previous month, participants were asked their reasons for choosing this preferred brand, including ‘I received it as a gift’ (others were ‘how they taste’ , ‘how good they make you feel’ , ‘this brand is less harmful to my health than other brands’ , ‘price’ , ‘high quality’ , the package’ and ‘it is a popular brand’).

#### Sociodemographics and smoking-related variables

Sociodemographic variables included age, sex, marital status, monthly household income (low=3000 Yuan or RMB or less; medium=3001-5000 RMB; high=5001 RMB or more), and education (low=elementary school or less; medium=junior high school and high school; high=college/university or more). Participants indicated one of 11 occupation categories, including farming, fishing and forestry workers, production operators, business or service industry workers, leaders of governments, Chinese Communist party organizations or companies, office clerks, professionals or specialized technicians, soldiers, students, retired, unemployed, and other occupations. Smokers were categorized into daily and less frequent smokers. Heaviness of smoking index (HSI) was calculated from cigarette consumption and the time elapsed from waking to smoking the first cigarette of the day, with a range of scores from 0 to 6 [32]. Participants were asked how long they had smoked their preferred brand (less than one year, 1 to 3 years, 4 to 5 years, 6 to 10 years, and more than 10 years).

### Analysis

The analyses were conducted using STATA, version 11, accounting for the sampling design and sampling weights. Pearson’s Chi-square tests were used to examine differences in gifting outcomes among participants with different characteristics. Logistic regression was used to estimate the bivariate and adjusted odds of gifting outcomes across sociodemographic and smoking-related variables.

## Results

### Sample characteristics

About 95% of smoker participants were males, consistent with nationally representative prevalence estimates in China [2]. Most were daily smokers (95%) and about 40% reported that they had smoked their preferred brand for less than three years (see Table 1 for more sample characteristics).

**Table 1:** Description of the sample characteristics

**Characteristics**		**All W2 Participants**		**Participants receiving most recent cigarettes as gifts**		**Participants choosing the preferred brand b/c gifts**	
		**N**	**% or Mean**	**N**	**% or Mean**	**N**	**% or Mean**
**City**	Beijing	801	23.4%	53	49.9%	69	33.8%
	Shenyang	799	13.5%	28	15.6%	57	14.9%
	Shanghai	803	28.1%	13	10.7%	19	10.5%
	Changsha	795	7.9%	23	4.9%	57	8.4%
	Guangzhou	833	14.6%	16	4.3%	78	21.7%
	Yinchuan	812	12.6%	33	14.5%	49	10.6%
**Age**	Average	4843	51.7	166	54.4	329	54.4
	Group 18-24	46	1.2%	0	0.0%	2	1.4%
	Group 25-39	766	14.9%	21	14.4%	48	16.9%
	Group 40-54	2352	47.4%	67	37.0%	126	31.2%
	Group 55+	1679	36.6%	78	48.6%	153	50.5%
**Sex**	Male	4589	96.0%	150	89.6%	305	93.6%
**Marital Status**	Married or living together	4356	89.1%	151	92.2%	289	85.9%
	Divorced, separated, widowed	304	6.4%	12	6.2%	25	10.0%
	Single	164	4.5%	2	1.7%	12	4.0%
**Education**	Low education level	588	11.9%	19	12.0%	56	20.4%
	Medium education level	3200	67.0%	85	52.2%	183	54.8%
	High education level	1025	21.2%	58	35.8%	86	24.8%
**Monthly household income**	Low income	794	14.4%	26	18.5%	62	17.4%
	Medium income	2199	48.5%	75	49.4%	137	49.9%
	High income	1524	37.1%	49	32.0%	109	32.7%
**Smoking frequency**	Everyday	4360	94.9%	125	76.2%	281	87.2%
	Some day	266	5.1%	41	23.8%	48	12.8%
**Heaviness of smoking index (HSI)**	Average	4457	2.37	159	1.41	311	2.02
	0	827	18.5%	67	41.4%	85	26.2%
	1	711	16.2%	28	18.3%	56	18.3%
	2	751	16.2%	23	11.3%	43	12.6%
	3	971	21.3%	24	19.8%	63	23.1%
	4	801	18.5%	10	5.4%	40	13.0%
	5	241	5.4%	6	3.3%	11	3.2%
	6	155	3.8%	1	0.5%	13	3.6%
**Years for smoking the preferred brand**	Less than one year	589	11.9%	44	28.0%	68	21.8%
	1 to 3 years	1226	26.4%	44	36.1%	92	31.3%
	4 to 5 years	675	15.1%	17	9.8%	36	10.9%
	6 to 10 years	709	15.8%	23	10.6%	33	9.8%
	More than 10 years	1348	30.7%	29	15.6%	88	26.3%

### Incidence and correlates of gifted cigarette acquisition

When asked about the source of their *most recently* obtained cigarettes, 3.5% of smokers (n=166) reported that they received them as gifts (see Table 2). Other sources were much more prevalent, particularly local stores, convenience stores or gas stations (40.7%), tobacco shops (24.9%), and supermarkets (23.3%).

**Table 2:** Sources of smokers’ most recently obtained cigarettes

**Source**	**Participants who reported the source of their most recent cigarettes**	
	**n**	**%**
Local store, convenience store, gas station	2,148	40.7%
Supermarket or hypermarket	1,034	23.3%
Tobacco shop	914	24.9%
Street vendor	281	6.0%
Gifts from others	166	3.5%
Other*	72	1.7%
Total	4615	100.0%

* For the percentage of the source was less than 1%, these sources were collapsed into one category. This category includes bars, cafeterias, tea bars, restaurants, hotels, duty-free shop, outside the country, military store, on the internet, vending machines, vender selling from a public transportation vehicle, others, and for the last year I didn’t buy cigarettes for myself.

Logistic regression models estimated the association between receipt of most recent cigarettes as a gift and both sociodemographic and smoking-related factors (see Table 3). Both bivariate and multivariate models indicated that smokers who were more than 55 years old, female, had college education or above, lived in Beijing, or smoked less often than daily were more likely to receive their most recent cigarettes as gifts than participants who were aged 25 to 39, male, had elementary education or under, lived in Shanghai and Guangzhou, or smoked daily, respectively. Participants who had smoked their preferred brand for more than one year and had a higher HSI were significantly less likely to receive their most recent cigarettes as gifts than those who had smoked their brand for less than one year and those who had a lower HSI, respectively. However, HSI was no longer statistically significant in the adjusted model.

**Table 3:** Factors associated with participants who received their most recent cigarettes as gifts and choosing their preferred brand based on cigarette received as gifts

**Covariate**	**Received their most recent cigarettes as gifts**						**Chose their preferred brand because of gifts**			
		**Bivariate**		**Multivariate**			**Bivariate**		**Multivariate**	
	**% row**	**OR**	**(95% CI)**	**AOR**	**(95% CI)**	**% row**	**OR**	**(95% CI)**	**AOR**	**(95% CI)**
City										
Beijing	7.5%	1				10.7%	1		1	
Shenyang	3.9%	0.65	(0.20, 1.27)	0.35	(0.12, 1.05)	7.8%	0.70	(0.37, 1.32)	0.62	(0.30, 1.26)
Shanghai	1.3%	0.21	(0.05, 0.49)**	0.20	(0.06, 0.66)**	2.5%	0.22	(0.16, 0.40)***	0.20	(0.08, 0.49)**
Changsha	2.2%	0.29	(0.11, 0.70)**	0.42	(0.18, 1.01)	7.4%	0.66	(0.37, 1.18)	0.77	(0.43, 1.41)
Guangzhou	1.0%	0.16	(0.04, 0.42)**	0.11	(0.03, 0.36)***	10.4%	0.97	(0.63, 1.48)	1.01	(0.60, 1.67)
Yinchuan	4.0%	0.53	(0.24, 1.11)	0.38	(0.15, 0.96)*	5.9%	0.52	(0.32, 0.84)**	0.48	(0.26, 0.88)*
Age										
Group 18-24	0.0%		(omitted)		(omitted)	7.8%	1		1	
Group 25-39	3.3%	1		1		7.8%	1.10	(0.28, 4.36)	2.03	(0.32, 12.76)
Group 40-54	2.7%	1.09	(0.62, 1.92)	1.45	(0.74, 2.84)	4.6%	0.75	(0.19, 2.99)	1.42	(0.21, 9.42)
Group 55+	4.7%	1.76	(1.04, 2.97)*	2.51	(1.44, 4.37)**	10.0%	1.63	(0.40, 6.65)	3.22	(0.47, 22.08)
Sex										
Male	3.2%	1		1		6.8%	1		1	
Female	9.5%	2.49	(1.33, 4.64)**	2.64	(1.13, 6.16)*	12.0%	1.73	(1.05, 2.85)*	1.28	(0.69, 2.36)
Marital status										
Married or living together	3.6%	1		1		6.7%	1		1	
Divorced, separated, widowed	3.3%	0.84	(0.40, 1.76)	0.87	(0.35, 2.19)	10.9%	1.71	(0.95, 3.05)	1.64	(0.86, 3.12)
Single	1.3%	0.31	(0.08, 1.25)	0.42	(0.06, 2.85)	6.1%	0.86	(0.37, 2.04)	1.22	(0.50, 3.00)
Income										
Low income	4.4%	1		1		8.6%	1		1	
Medium income	3.5%	0.90	(0.54, 1.51)	0.72	(0.38, 1.34)	7.2%	0.84	(0.52, 1.35)	0.84	(0.50, 1.43)
High income	3.0%	0.87	(0.35, 2.14)	0.59	(0.22, 1.58)	6.3%	0.81	(0.49, 1.33)	0.81	(0.44, 1.46)
Education										
Low education level	3.5%	1		1		12.5%	1		1	
Medium education level	2.7%	0.88	(0.42, 1.80)	1.15	(0.54, 2.46)	5.7%	0.50	(0.35, 0.73)***	0.63	(0.43, 0.92)*
High education	5.8%	2.07	(1.05, 4.09)*	2.81	(1.33, 5.94)**	8.2%	0.71	(0.47, 1.07)	0.93	(0.60, 1.44)
Smoking frequency										
Everyday	2.8%	1		1		6.4%	1			
Some day	16.0%	6.38	(4.44, 9.17)***	3.67	(2.45, 5.50)***	17.5%	3.01	(1.90, 4.77)***	2.43	(1.46, 4.03)**
Heaviness of smoking index (HSI)										
0	7.7%	1		1		9.7%	1		1	
1	3.9%	0.56	(0.33, 0.96)*	0.89	(0.51, 1.53)	7.8%	0.85	(0.49, 1.48)	1.08	(0.61, 1.91)
2	2.4%	0.29	(0.17, 0.50)***	0.48	(0.27, 0.85)*	5.3%	0.60	(0.34, 1.04)	0.67	(0.40, 1.12)
3	3.2%	0.37	(0.21, 0.65)**	0.56	(0.32, 0.99)*	7.5%	0.75	(0.49, 1.15)	0.87	(0.54, 1.41)
4	1.0%	0.13	(0.07, 0.22)***	0.24	(0.14, 0.41)***	4.8%	0.51	(0.29, 0.89)*	0.58	(0.37, 0.92)*
5	2.1%	0.23	(0.10, 0.54)**	0.40	(0.14, 1.15)	4.0%	0.40	(0.18, 0.91)*	0.49	(0.22, 1.10)
6	0.4%	0.03	(0.00, 0.24)**	0.07	(0.01, 0.56)*	6.5%	0.66	(0.32, 1.34)	0.71	(0.35, 1.45)
Years for smoking the brand most										
Less than one year	7.9%	1		1		12.4%	1		1	
1 to 3 years	4.6%	0.50	(0.29, 0.86)*	0.45	(0.25, 0.83)*	8.0%	0.69	(0.48, 0.99)*	0.68	(0.47, 0.99)*
4 to 5 years	2.2%	0.26	(0.10, 0.69)**	0.16	(0.05, 0.45)**	4.9%	0.46	(0.24, 0.89)*	0.41	(0.20, 0.83)*
6 to 10 years	2.3%	0.31	(0.15, 0.63)**	0.28	(0.11, 0.73)**	4.2%	0.39	(0.24, 0.62)***	0.42	(0.25, 0.70)**
More than 10 years	1.7%	0.19	(0.11, 0.35)***	0.16	(0.08, 0.32)***	5.8%	0.61	(0.35, 1.07)	0.59	(0.33, 1.05)

Significant levels for logistic regression: *p<0.05; **p<0.01; ***p<0.001.

### Prevalence and correlates of choosing current preferred brand because it was gifted

About 7.0% of smokers (n=329) reported that they chose their preferred brand because it had been received as a gift. The majority of smokers reported that their decision in choosing their preferred brand was based on that brand’s taste (73.3%), price (67.2%), high quality (62.4%), and how good that cigarette brand made them feel (61.1%) (Table 4). Bivariate and multivariate models showed that participants who lived in Shanghai and Yinchuan, were high school graduates, smoked daily, or smoked the preferred brand for one to ten years were less likely to choose their preferred brand because it was gifted than those who lived in Beijing, had elementary education or under, did not smoke daily, or smoked the preferred brand less than one year, respectively (Table 3).

**Table 4:** Smokers’ reasons for choosing their preferred brand*

**Reason**	**W2 participants who chose their preferred brand**	
	**n**	**%**
Taste	3,469	73.3%
Price	3,194	67.2%
High quality	2,980	62.4%
Feel good	2,957	61.1%
Less harmful to my health	1,776	38.2%
Popular brand	1,519	32.3%
Package	1,278	28.4%
Gifts from others	329	7.0%

* Participants were asked their decision in choosing the brand they smoked most separately for each reason.

## Discussion

This population-based study found that 3.5% of adult smokers in six Chinese cities received their *most recent* cigarettes as a gift. This estimate is substantially lower than estimates in previous surveys (ranging from 53% to 60%) [21,22]. But those other surveys had asked smokers whether they had received cigarettes as a gift at any time over a much longer time frame (e.g., previous three months or previous six months). It is possible, however, to compare the incidence of cigarette gifts at the most recent procurement with the estimated incidence of receiving a cigarette gift at least once over a longer period. In our sample, the number of cigarettes smoked per day and the average number of packs or cartons most recently purchased suggests that smokers would purchase cigarettes an average of 2.8 times per week. If receipt of cigarettes as gifts is randomly distributed in the population, the probability of a smoker receiving cigarettes as gifts would be 0.73 in the previous 3 months (number of weeks = 13), and 0.35 if the time frame was in the past month (number of weeks = 4.3), using the formula P = 1-(1–0.035)^(number of weeks of the time period x 2.8 purchases/week). The receipt of gifts is unlikely to be random and so this formula likely overestimates the prevalence of cigarette gift receiving. However, it suggests some consistency between our estimation of incidence and previous prevalence estimates. Hence, our study confirms that the prevalence of smokers receiving cigarettes as gifts is high and cause for concern.

There is another way of understanding the implications of our findings that is not dependent on the non-randomness of who receives gifts. This is by focusing on the frequency of receiving cigarettes as gifts. If there is a .035 probability of receiving cigarettes as gifts, which implies that this occurs once every 1/.035 = 28.6 times that a smoker obtains cigarettes. Given the average rate of purchasing/obtaining cigarettes is 2.8 times per week, that would further imply that the average smoker in China receives a gift of cigarettes once every 28.6/2.8 = 10.2 weeks, that is, about five times a year (because of clustering, some smokers will receive gifts more often than others, but the average across all smokers will be once every 10.2 weeks).

It is noteworthy that the data collection period for our study did not significantly overlap with gift-giving festivals, such as Lunar New Year (February 7, 2008) and Mid-Autumn Festival (September 25, 2007) — only 2.3% (n=113) of participants were interviewed within 15 days before and after these two holidays. The 2.3% of participants represented a very small proportion (n=4) of the 3.5% who reported receiving the most recent cigarettes as gifts. After excluding participants who were interviewed around the two holidays, the incidence of receiving cigarette gifts remains similar (3.5%). Other studies have been conducted during times when the reporting period would contain these important periods in which gifts (of any kind) are very common. This difference in time of year between the present study and those of past studies likely contributes further to lower reported incidence of receiving cigarettes as gifts.

Our findings showed that the self-reported influence of receiving cigarettes as gifts on brand selection was relatively low, especially when considered alongside other reasons for brand selection (i.e., taste, price, quality). However, these data may underestimate the influence that gifts may have on brand selection due to recall issues, as shown by higher percentages of gifting influences found among those participants who smoked their preferred brand for less than one year (7.9%). Participants may have been more likely to remember taste and other factors that continue to provide a conscious influence over the smoking experience. Another reason why there may be a low linkage between receiving cigarettes as gifts and current brand is because many gift givers may well know the brand choice of the recipient and will choose that brand to give; in such situations, the smoker would report that receiving the gift was not a factor in their brand choice because brand choice had preceded the gift. Future studies should focus only on smokers who had switched brands, and examine the probability that a gift was mentioned as a reason for switching brands.

Compared to smokers in other cities, a significantly higher proportion of smokers in Beijing reported that they received their most recent cigarette as gifts (7.5% vs. 1.0 to 4.0%). This may be due to the high concentration of central governmental offices and business headquarters in Beijing, as people who work and interface with these settings appear more likely to practice cigarette gifting [3]. Shanghai smokers were significantly less likely to report that they chose their preferred brand based on cigarette gifts than Beijing smokers, perhaps partly because Shanghai smokers had the lowest incidence of receiving cigarette gifts. However, Guangzhou participants had the second highest proportion of choosing the preferred brand based on cigarette gifts despite the lowest incidence of receiving cigarette gifts among six cities, which is consistent with Guangzhou’s relatively lower prevalence of giving cigarettes as gifts in previous study [30]. Other unmeasured differences in characteristics of smokers across cities, such as specific meanings of cigarette gifts may also have influenced our findings and merit further research, particularly if tobacco control strategies are developed to change these meanings.

One striking finding is that female smokers were more likely than males to report receiving current cigarettes as gifts and to have chosen their preferred brand because of gifting, which suggests that social norms discouraging female smoking may be changing. Previous studies have focused on cigarette gifting among Chinese males perhaps because of the very low prevalence of female smoking (3%) [4,19,27]. It is notable that Chinese tobacco companies have started marketing attractive cigarette products and gift packaging that deliberately targets female smokers [33]. The greater prevalence of gifting and its influence on brand selection among women deserve further investigation into how social and gender norms around smoking may be changing.

Participants older than 55 years were more likely than young smokers to report receiving cigarette gifts, which likely reflects customs around gifting to elders, particularly fathers and fathers-in-laws, to show respect [4,5]. Most participants in this age group were retired, indicating that occupation does not appear to drive cigarette gifting in this group. Participants with high education were also more likely to report receiving recent cigarette gifts than those with low education, which may also reflect differences in social status. Nevertheless, participants with low education were more likely to report that they chose their preferred brand because of cigarette gifts than participants with mid-level education. This subgroup of lowest educational attainment may pursue status symbols presented by cigarette gift brands to augment their self image [3,4].

It is noteworthy that smokers who smoked daily and had higher HSI were less likely to report they received their most recent cigarette as gifts. Heavy smokers must purchase cigarettes more often to meet their consumption needs than people who smoke less regularly. The higher frequency of purchasing cigarettes from all kinds of sources would reduce chances that receiving the most recent cigarette would be a gift, whereas lighter smokers will have a pack for a longer period of time. To better understand the association between receiving cigarettes as gifts and characteristics of cigarette gift receivers, future research should ask smokers about receiving cigarette gifts over different periods of time. A more refined operationalization of measurement could better capture some of the nuances around this practice across subgroups who smoke at different frequencies.

Participants who smoked daily and had higher HSI were also less likely to report cigarette gifts were part of their decision to choose their preferred brand. There could be various reasons for this. Cigarette gifts may not provide a compelling reason for heavy smokers to switch brands because of inveterate smoking and purchasing habits due to their high nicotine dependency and high brand loyalty. Heavy smokers tend to buy less expensive cigarettes [34,35] because they may bear more financial burden from their high demand of tobacco use than light smokers, so they are less likely to choose cigarette gift brands as the preferred brand since cigarette gifts are usually expensive [4,29]. Price concern may also explain that smokers who chose the preferred brand because of gifts were less likely to smoke that brand more than one year. Smokers who are sensitive to cigarette price are more likely to switch to cheaper brands to maintain their smoking habit [36], thus being less loyal to specific brands over time.

Interpretation of our results is limited by a number of issues. For example, young smokers were undersampled, especially in the age group 18–24, due to their absence from sampled households because they were at school or work; however, our data suggest that this group is less likely than older groups to receive cigarettes as gifts. Smokers’ report of choosing their preferred brand because someone gave it to them as a gift may underestimate the real prevalence, due to recall bias and to competing more apparent preference factors, such as taste and quality. Because of the cross-sectional survey design, our data is unable to make causal links. The incidence of receiving cigarette gifts was derived from the measure used to assess the source of the most recent cigarettes that smokers obtained/purchased and was also affected by the timing of data collection, capturing a smaller sample of this subgroup. Future studies that measure gift receipt across a variety of reference periods and that collect data at different times of the year may be necessary to better estimate and monitor the prevalence of receiving cigarette gifts. The response categories for occupation did not appear to allow for the level of discernment necessary to adequately explore the role of occupation, as many smokers did not provide responses that fit within the given response options. Future studies should include more specific occupational categories, especially those that appear most highly associated with cigarette gifting, such as doctors [13,19]. In addition to brand selection, the impact of receiving cigarettes gifts on smokers’ quit intentions, attempts or smoking relapse should be examined with a longitudinal cohort study design since it is one of major reasons that Chinese smokers find it hard to give up smoking [16,29].

## Conclusions

This study provides the first population-based estimates of the incidence and correlates of cigarette gift receiving in China outside of major festival periods, as well as the self-reported effects that gifting has on brand selection. Future research should examine whether the same types of people receive cigarettes as gifts at other times of the year, such as the Lunar Festival. Graphic warning labels on cigarette packages and mass media campaigns that directly address this issue may be necessary to reduce the social acceptability of giving cigarettes as gifts [37,38], as the tobacco industry actively works to integrate its brands into this deeply-rooted Chinese tradition.

## Competing interests

The authors declare that they have no competing interests.

## Authors’ contributions

LH devised the study, conducted the analyses, and oversaw the writing of the manuscript. JFT helped develop the analysis plan and helped wrote the manuscript. GTF, QL, ACKQ, and YJ, contributed to the manuscript by providing key information on the study context, suggestions about the analysis approach, and interpretation of results. All authors read, provided comments, and approved the final manuscript.

## Pre-publication history

The pre-publication history for this paper can be accessed here:

http://www.biomedcentral.com/1471-2458/12/996/prepub
